# Antibiotic Therapy Does Not Alter the Humoral Response to Vaccination for Porcine Circovirus 2 in Weaned Pigs

**DOI:** 10.3390/vetsci6020051

**Published:** 2019-05-30

**Authors:** Jonathan E. Fogle, Jenna A. Scott, Glen W. Almond

**Affiliations:** College of Veterinary Medicine, Department of Population Health and Pathobiology, North Carolina State University, 1052 William Moore Dr, Raleigh, NC 27606, USA; jefogle@ncsu.edu (J.E.F.); jascott7@ncsu.edu (J.A.S.)

**Keywords:** Pig, vaccine, antibiotic

## Abstract

Recent reports suggest that antibiotic therapy may either reduce or enhance the immune response to various porcine vaccines. Based upon these findings, we asked if antibiotic therapy alters immune cell populations, as measured by flow cytometry and/or vaccine-specific humoral immunity, as measured by sample to positive (S/P) antibody ratios. Here, we investigated the immuno-modulatory effects of enrofloxacin, ceftiofur, and tulathromycin on the immune response to a *Mycoplasma hyopneumoniae* (*M. hyopneumoniae*) and porcine circovirus type 2 (PCV-2) combination vaccine in weaned pigs. Maternal antibody likely interfered with the induction of immunity to *M. hyopneumoniae.* Antibiotic administration did not affect immune cell populations, as assessed by flow cytometry and did not affect the induction of humoral immunity to PCV-2.

## 1. Introduction

In the commercial swine industry, pigs are commonly vaccinated and administered antibiotics simultaneously [1,2,3,4]. With this production practice, it is assumed that no interactions occur between the vaccination and antibiotic therapy. Recent reports, however, demonstrate that antibiotics may modulate the immune response to vaccines when antibiotics and vaccines are administered simultaneously in 8- to 12-week-old pigs [1,2,3,4]. One study reported that enrofloxacin negatively impacts both the cell-mediated and humoral post-vaccinal immune responses, as measured by cytokine and antibody production, respectively [1]. Two additional studies demonstrated that ceftiofur also negatively impacts the cell-mediated and humoral responses to vaccines [2,4]. However, other antibiotics, including doxycycline and tulathromycin, are reported to have either no significant impact or to enhance the humoral response to vaccines [3,4]. Collectively, these reports indicate that more information is needed regarding the potential immuno-modulatory action of antibiotics in post-weaned pigs and their effects upon vaccine responses. Therefore, the objective of this study was to determine if enrofloxacin, tulathromycin, and ceftiofur altered immune cell populations as assessed by complete blood count (CBC) and flow cytometry and/or antigen-specific IgG antibody production in weaned pigs vaccinated with a PCV-2/*M. hyopneumoniae* combination vaccine.

## 2. Materials and Methods

The animal use protocol for this study was approved by the North Carolina State University Institutional Animal Care and Use Committee (protocol #19-052-T). The study was conducted in a university teaching unit using pigs with a genetic background typical to the production animals used regionally. Treatments included unvaccinated control pigs (CON), a PCV-2/*M. hyopneumoniae* vaccine (Fostera® PCV MH) only (VAX), tulathromycin only (TUL), ceftiofur only (CEFT), enrofloxacin only (ENRO) or the combination of the PCV-2/*M. hyopneumoniae* vaccine with the antibiotics (VAX + TUL, VAX + CEFT, VAX + ENRO). Pigs (n = 64) were selected at weaning (approximately 21 days of age; day 0 of the study), identified with an ear tag and randomly assigned to 8 treatment groups (n = 8/group, n = 7 in VAX + ENRO). One pig died before completion of the study and was removed from the data analysis. Vaccinations were administered as a 2 mL dose on day 0 via an intramuscular (IM) injection in the left side of the neck. Antibiotics were also administered on day 0 via an IM injection in the right side of the neck with the appropriate volume and dose adjusted for pig body weight (BW). Specifically, the tulathromycin product (Draxxin®25) was given at a dose of 2.5 mg/kg BW or 1.0 mL/22 lbs BW. The ceftiofur product (Excede®) was given at 2.27 mg ceftiofur/lbs BW or 1.0 mL/44 lbs BW while the enrofloxacin product (Baytril®100) was administered as a single dose at 7.5 mg/lbs BW. Pigs not receiving antibiotics were injected IM with saline in the right side of the neck. Body weights and blood samples were collected on study days 0 (prior to vaccination), 14, 21, and 35 for CBC, serum ELISAs, and flow cytometry. Serum samples were submitted to the Iowa State University Diagnostic Laboratory to determine S/P ratios against *Mycoplasma hyopneumoniae* and porcine circovirus type 2 using two commercially available ELISA kits—Ingezim Circovirus IgG (eurofins®) and the Mycoplasma Hyopneumoniae Antibody Test Kit (IDEXX®), respectively—to measure antigen-specific IgG. Whole blood samples were processed for flow cytometric analysis of lymphocyte subsets using a previously established protocol [5]: Series 1: CD8α^+^ T cells (cytotoxic T lymphocytes (CTLs); CD8α, mouse IgG_2a_, MCA1223PE, AbD Serotec, Oxford, UK) at 1:50 dilution per tube with R-PE; and CD4^+^ T cells (CD4α, mouse IgG_2b_, MCA1749F, AbD Serotec, Oxford, UK) at 1:100 dilution per tube with FITC. Series 2: CD3^+^ T cells (CD3e, mouse IgG_2b_κ, BB238E6, Southern Biotech, Birmingham, AL) at 1:50 dilution per tube with FITC; B cells (CD21, mouse IgG_1_κ, BB6-11C9.6, Southern Biotech, Birmingham, AL) at 1:200 dilution per tube with R-PE. Approximately 20,000 events were acquired using a Becton Dickinson LSRII flow cytometer and analyzed using FACS Diva software. Statistical analysis included repeated measures ANOVA with treatment, time (age of pig) and treatment/time interaction used as factors. The sow was included as a covariate and random effect while the measurement of response at time 0 was a fixed effect. Least square means were estimated and compared between treatments by time, between treatments, overall and between time points within each treatment. Unadjusted *p*-values and adjusted *p*-values (Tukey’s method) were determined. All statistical measurements were performed in collaboration with our college biostatistical consulting service.

## 3. Results

There was no difference in CBC parameters when treatment groups were compared (not shown). As reported in Table 1, we observed an increase in the absolute lymphocyte count with increasing age, within each group, at days 14, 21, and 35. For the various immune cell populations, there was no significant effect of antibiotic administration on the number or percentage of lymphocytes at days 0, 14, 21, and 35. The mean and standard error of the mean (SEM) for lymphocyte subset percentages at day 35 are reported in Table 2.

**Figure 1:**
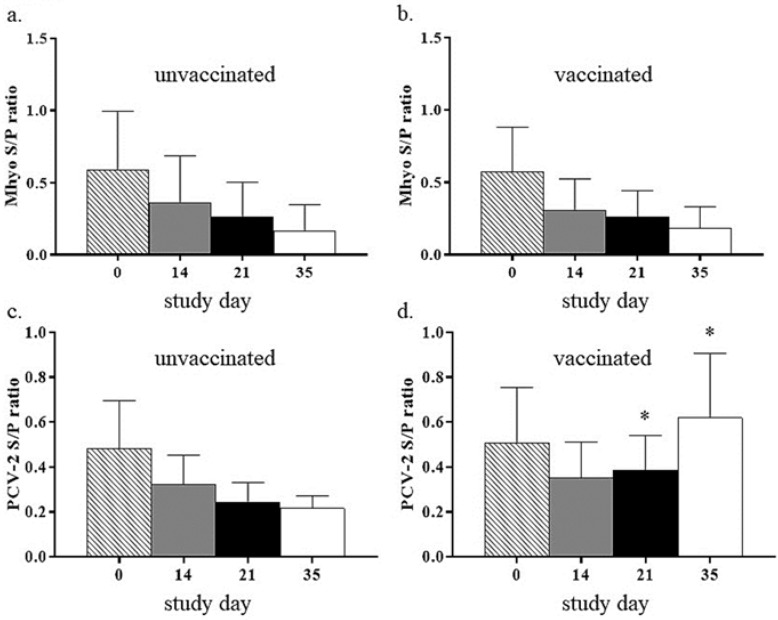
Sample to positive (S/P) ratios to *Mycoplasma hyopneumoniae (Mhyo)* and porcine circovirus type 2 (PCV-2). Pigs were either unvaccinated (n = 31) or vaccinated with a *Mhyo*/PCV-2 vaccine (n = 32) prior to weaning. (**a**,**b**). For both unvaccinated and vaccinated groups, the *Mhyo* S/P ratios declined over time. (**c**). For the PCV-2 unvaccinated groups, the PCV-2 S/P ratio declined over time. (**d**). For the PCV-2 vaccinated groups, the PCV-2 S/P ratio declined and then rebounded over time. (**c**,**d**). For days 21 and 35 the PCV-2 S/P ratio was higher when compared to unvaccinated controls (*p* < .05, asterisks).

**Figure 2:**
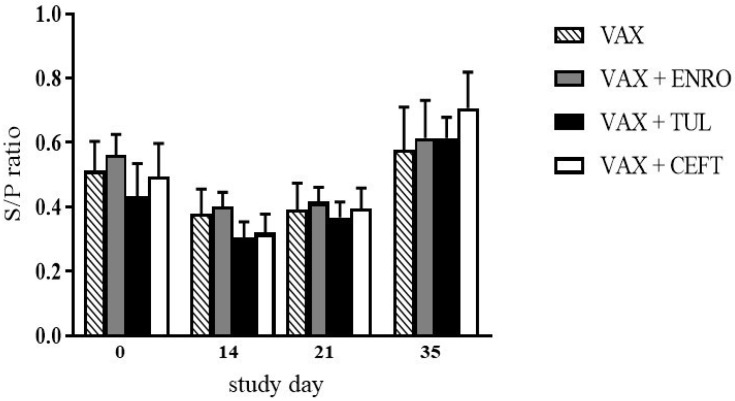
Assessment of PCV-2 S/P ratio over time for weanling pigs administered different antibiotics. There was no difference in PCV-2 S/P ratios when vaccinated pigs (VAX, diagonal bars) were compared to vaccinated pigs administered the three different antibiotics (n = 8 pigs per antibiotic treatment group, solid bars, see legend, each bar represents mean + SEM).

Figure 1 compares *M. hyopneumoniae* and PCV-2 S/P ratios between vaccinated (VAX, VAX + ENRO, VAX + CEFT, and VAX + TUL groups) and unvaccinated (CON, ENRO, CEFT, and TUL groups) pigs throughout the duration of the study. In unvaccinated pigs, ratios against both of the pathogens were highest at day 0 and lowest at day 35. In all pigs receiving the PCV-2/*M. hyopneumoniae* vaccine, ratios against *M. hyopneumoniae* also decreased throughout the study. However, all pigs receiving the vaccine had increased PCV-2 S/P ratios at day 35. Further, there was a significant difference (*p* < .05, asterisks) in PCV-2 S/P ratios between vaccinated and unvaccinated pigs at days 21 and 35 of the study. Figure 2 compares PCV-2 S/P ratios between pigs in the VAX, VAX + ENRO, VAX + CEFT, and VAX + TUL groups. In each group, PCV-2 ratios had similar kinetics, and there was no statistical significance (*p* > .05) among the treatment groups at all time points. 

## 4. Discussion

The purpose of this study was to evaluate the potential immuno-modulatory effects of the antibiotics ceftiofur, tulathromycin, and enrofloxacin on the immune response to a PCV-2/*M. hyopneumoniae* vaccine in pigs at weaning. We asked two specific questions: (1) Does antibiotic administration alter immune cell subsets and (2) does antibiotic administration alter vaccine-specific humoral immunity? The postvaccinal immune response was assessed via CBCs, flow cytometric analysis of lymphocyte subsets, and measurement of S/P ratios against PCV-2 and *M. hyopneumoniae*. In the study presented here, no significant differences were detected between treatment groups in CBC or flow cytometry results. Instead, the only factor affecting immune cell populations appeared to be the age of the pig. This contrasts with one study which reported a reduction in the percentage and absolute number of T cells in pigs vaccinated for pseudorabies and concurrently administered doxycycline in the drinking water [3]. This difference may be explained by the use of a different vaccine and/or the age of the pigs in relation to vaccine administration. In the current study, pigs were vaccinated at three weeks of age while pigs in the doxycycline study were vaccinated at eight and 10 weeks of age for the first and second doses of the pseudorabies virus vaccine. 

Pigs in all treatment groups had decreasing S/P ratios against *M. hyopneumoniae* throughout the duration of the study, regardless of whether they were vaccinated or not. This may have been due to a delay in response to the *M. hyopneumoniae* vaccine or inactivation by maternal antibody. For example, Park et al. reported that 21-day-old, seronegative pigs vaccinated with a PCV-2/*M. hyopneumoniae* vaccine did not seroconvert for *M. hyopneumoniae* following vaccination; but seroconverted following inoculation with *M. hyopneumoniae* 21 days after vaccine administration [6]. The results reported by Park et al. may explain the lack of response to the *M. hyopneumoniae* component in the study presented here. However, given the age of the pigs (21 days), it is most likely that these were maternal antibodies. In contrast, vaccinated pigs in the current study had an increase in PCV-2 S/P ratios between days 21 and 35 of the study, indicating a response to the killed PCV-2 virus in the vaccine. We postulate that maternal antibodies “masked” the *M. hyopneumoniae* bacterin component of the vaccine; meaning that the bacterin was coated with maternal antibody and eliminated prior to inducing an immune response in the weaned pig. In contrast, the killed PCV-2 appeared able to induce humoral immunity, but the differences in antigenicity between the two components are not entirely clear. Limitations of the study included the inability to assess antibiotic therapy on the humoral response to *M. hyopneumoniae*, the duration of the study (no samples were collected past day 35), and a relatively small sample size which potentially masked the effects of vaccination and/or antibiotic administration.

When comparing anti-PCV-2 antibody responses between vaccinated pigs that did and did not receive an antibiotic, kinetics were similar across the VAX, VAX + ENRO, VAX + TUL and VAX + CEFT groups (Figure 2). Therefore, there was no evidence that the antibiotics tested in this study modulated the immune response to the PCV-2 portion of the vaccine. This is contrary to previous studies reporting that enrofloxacin and ceftiofur decreased the humoral response to live-attenuated pseudorabies virus, inactivated swine influenza virus, and inactivated erysipelas vaccines [1,2,4]. Further, another study observed that tulathromycin increased the humoral response to an inactivated erysipelas vaccine [4]. The discrepancies between the study results may be explained by the use of different vaccines, as the immuno-modulatory effect of antibiotics on the humoral postvaccinal immune response are likely dependent upon the vaccine antigen components, including live vs. killed and vaccine adjuvant components [2,3]. 

In summary, pigs are commonly administered antibiotics and vaccines simultaneously within the commercial swine industry and previous studies have shown that antibiotics may modulate the immune system—hindering or enhancing the immune responses to vaccines. With the basic immune parameters assessed here, we found no evidence to support antibiotic immuno-modulation to PCV-2 vaccination in recently weaned, three-week-old pigs. 

## Figures and Tables

**Table 1:** Mean total lymphocyte counts for each treatment group at days 0, 14, 21, and 35. Unvaccinated control animals (CON), vaccination only (VAX), tulathromycin only (TUL), ceftiofur only (CEFT), enrofloxacin only (ENRO) or the combination of the PCV-2/*M. hyopneumoniae* vaccine with the antibiotics (VAX + TUL, VAX + CEFT, VAX + ENRO). There was no difference when each group was compared to each other at days 0, 14, 21, and 35. However, there was a significant difference within each group over time (days 14, 21, and 35, p < .05, asterisks).

	Day 0	Day 14 *	Day 21 *	Day 35 *
CON	4977	6887	8391	8628
VAX	4905	6951	8443	7837
TUL	4879	6272	7520	9147
CEFT	4646	6835	6975	10,074
ENRO	5176	5989	7105	8989
VAX + TUL	4495	6347	7389	8422
VAX + CEFT	4454	7041	7723	8141
VAX + ENRO	4351	6153	6098	7397

* p < .05.

**Table 2:** The percentage of lymphocyte subsets at day 35. The percentage of lymphocyte subsets in peripheral blood was assessed by flow cytometry. The mean and SEM (parentheses) were recorded for each lymphocyte subset. There was no difference in immune cell percentages between treatment groups. Additionally, there was no difference between groups at days 0, 14, 21 (not shown).

Day 35	% CD21+	% CD3+	% CD4+	% CD8+	% CD4+CD8+
CON	15.5 (1.6)	44.0 (2.0)	27.7 (2.0)	12.4 (2.3)	1.5 (0.6)
VAX	15.9 (1.7)	45.8 (2.5)	25.3 (2.0)	13.5 (2.4)	0.3 (.07)
TUL	15.2 (1.7)	45.0 (2.7)	22.5 (2.8)	13.1 (1.7)	1.7 (0.8)
CEFT	13.2 (1.5)	45.7 (3.5)	23.6 (2.6)	16.4 (3.1)	0.1 (0.3)
ENRO	12.6 (1.8)	46.8 (2.9)	20.0 (2.0)	16.3 (2.4)	0.8 (0.1)
VAX + TUL	14.7 (2.0)	47.2 (1.9)	26.2 (2.0)	13.1 (2.3)	0.8 (0.2)
VAX + CEFT	15.1 (1.8)	46.4 (4.0)	25.0 (2.1)	16.9 (2.9)	0.7 (0.2)
VAX + ENRO	15.2 (1.6)	42.3 (2.1)	22.8 (3.6)	14.9 (3.2)	0.9 (0.3)

## References

[1] Pomorska-Mól M., Czyzewska-Dors E., Kwit K., Rachubik J., Lipowski A., Pejsak Z. (2015). Immune response in pigs treated with therapeutic doses of enrofloxacin at the time of vaccination against Aujeszky’s disease. Res. Vet. Sci..

[2] Pomorska-Mól M., Czyzewska-Dors E., Kwit K., Wierzchoslawski K., Pejsak Z. (2015). Ceftiofur hydrochloride affects the humoral and cellular immune response in pigs after vaccination against swine influenza and pseudorabies. BMC Vet. Res..

[3] Pomorska-Mól M., Kwit K., Markowska-Daniel I., Pejsak Z. (2014). The effect of doxycycline treatment on the postvaccinal immune response in pigs. Toxicol. Appl. Pharmacol..

[4] Pomorska-Mól M., Kwit K., Wierzchoslawski K., Dors A., Pejsak Z. (2016). Effects of amoxicillin, ceftiofur, doxycycline, tiamulin and tulathromycin on pig humoral immune responses induced by erysipelas vaccination. Vet. Rec..

[5] Kick A., Tompkins M., Whisnant S., Flowers W., Almond G.W. (2012). Effects of weaning age on the adaptive immune system in pigs. J. Anim. Sci..

[6] Park C., Jeong J., Choi K., Chae C. (2016). Efficacy of a new bivalent vaccine of porcine circovirus type 2 and Mycoplasma hyopneumoniae (FosteraTM PCV MH) under experimental conditions. Vaccine.

